# Whole genome sequencing provides possible explanations for the difference in phage susceptibility among two *Salmonella* Typhimurium phage types (DT8 and DT30) associated with a single foodborne outbreak

**DOI:** 10.1186/s13104-015-1687-6

**Published:** 2015-11-27

**Authors:** Manal Mohammed, Martin Cormican

**Affiliations:** School of Medicine, National University of Ireland Galway, Galway, Ireland

**Keywords:** *Salmonella* Typhimurium, Phage typing, DT8 and DT30, Comparative genomics

## Abstract

**Background:**

Phage typing has been used for decades as a rapid, low cost approach for the epidemiological surveillance of *Salmonella enterica* subsp. *enterica* serovar Typhimurium. Although molecular methods are replacing phage typing the system is still in use and provides a valuable model for study of phage-host interaction. Phage typing depends on the pattern of bacterial resistance or sensitivity to a panel of specific bacteriophages. In the phage typing scheme, *S.* Typhimurium definitive phage types (DT) 8 and 30 differ greatly in their susceptibility to the 30 typing phages of *S.* Typhimurium; DT8 is susceptible to 11 phages whereas DT30 is resistant to all typing phages except one phage although both DT8 and DT30 were reported to be associated with a single foodborne salmonellosis outbreak in Ireland between 2009 and 2011. We wished to study the genomic correlates of the DT8 and DT30 difference in phage susceptibility using the whole genome sequence (WGS) of *S.* Typhimurium DT8 and DT30 representatives.

**Results:**

Comparative genome analysis revealed that both *S.* Typhimurium DT8 and DT30 are lysogenic for three prophages including two *S.* Typhimurium associated prophages (Gifsy-2 and ST64B) and one *S.* Enteritidis associated prophage (Enteritidis lysogenic phage S) which has not been detected previously in *S.* Typhimurium. Furthermore, DT8 and DT30 contain identical clustered regularly interspaced short palindromic repeats (CRISPRs). Interestingly, *S.* Typhimurium DT8 harbours an accessory genome represented by a virulence plasmid that is highly related to the pSLT plasmid of *S.* Typhimurium strain LT2 (phage typed as DT4) and codes a unique methyltransferase (MTase); M.EcoGIX related MTase. This plasmid is not detected in DT30. On the other hand, DT30 carries a unique genomic island similar to the integrative and conjugative element (ICE) of Enterotoxigenic *Escherichia coli* (ETEC) and encodes type IV secretion pathway system (T4SS) and several hypothetical proteins. This genomic island is not detected in DT8.

**Conclusions:**

We suggest that differences in phage susceptibility between DT8 and DT30 may be related to acquisition of ICE in DT30 and loss of pSLT like plasmid that might be associated with DT30 resistance to almost all phages used in the typing scheme. Additional studies are required to determine the significance of the differences among DT8 and DT30 in relation to the difference in phage susceptibility. This study represents an initial step toward understanding the molecular basis of this host-phage relationship.

**Electronic supplementary material:**

The online version of this article (doi:10.1186/s13104-015-1687-6) contains supplementary material, which is available to authorized users.

## Background

Salmonellosis is an infectious disease affecting humans and almost all known animals. It is caused by members of the genus *Salmonella*. *Salmonella enterica* subsp. *enterica* serovar Typhimurium is a leading cause of foodborne illness worldwide. Routine epidemiological surveillance of *S.* Typhimurium infections has been performed for decades using *S.* Typhimurium phage typing scheme [1]. Phage typing depends on the pattern of bacterial resistance or sensitivity to a panel of *S.* Typhimurium specific bacteriophages. More than 300 definitive phage types (DTs) are recognised [2]. However, the evolutionary relationship among different phage types is not well described. Bacteria may have several antiviral mechanisms associated with resistance to infection by bacteriophages. These include masking the surface receptors by capsule or other surface components to block phage adsorption to the host cell [3]. Other systems within the bacteria are also involved including restriction modification (RM), clusterd regulatory interspaced short palindromic repeats (CRISPR) loci coupled to CRISPR associated sequence (CAS) proteins and superinfection exclusion (Sie) systems [4, 5].

Phage typing has proved to be very useful in investigating *S.* Typhimurium foodborne outbreaks. Two large *S.* Typhimiurium DT8 outbreaks associated with consumption of duck eggs were reported in Europe. In 2010 an outbreak of *S.* Typhimurium DT8 was reported in England and Northern Ireland [6]. Another outbreak of *S.* Typhimurium DT8 was reported in Ireland over a 19-month period between August 2009 and February 2011 [7]. The outbreak was also linked to the consumption of duck eggs where *S.* Typhimurium DT8 and DT30 were isolated. DT8 was more predominant during the outbreak and was isolated from humans and duck eggs however, DT30 were isolated from ducks during the outbreak (but non from humans). *S.* Typhimurium DT8 and DT30 are considered closely related but differ greatly in phage susceptibility. *S.* Typhimurium DT8 is susceptible to typing phage 8 and to varying degrees to 10 of 30 whereas DT30 is susceptible only to typing phage 8 as illustrated in Table 1. Here, we report genomic differences between DT8 and DT30 that may be relevant to this difference in phage susceptibility.

**Table 1:** Pattern of reaction to typing phage set in *S.* Typhimurium DT8 and DT30 in addition to DT1, DT4 and DT44

Phage type	Result for indicated phage																													
	1	2	3	4	5	6	7	8	10	11	12	13	14	15	16	17	18	19	20	21	22	23	24	25	26	27	28	29	32	35
DTB	–	–	–	–	–	–	–	CL	SCL	++/SCL	–	–	–	–	+++	–	–	–	SCL	–	SCL	SCL	–	±	±	–	–	CL	CL	–
DT30	–	–	–	–	–	–	–	CL	–	–	–	–	–	–	–	–	–	–	–	–	–	–	–	–	–	–	–	–	–	–
DT4	–	–	–	CL	CL	CL	–	–	SCL	OL	++	++	–	CL	CL	–	–	CL	CL	±	CL	CL	–	SCL	+	CL	–	CL	CL	CL
DT1	CL	CL	CL	CL	CL	CL	CL	–	+++	CL	CL	CL	CL	CL	CL	CL	CL	CL	+++	CL	CL	CL	CL	CL	CL	CL	∓	CL	+++	CL
DT44	+/+++	+++	+++	–	–	–	SCL	–	+++	+/+++	–	–	CL	–	+++	+++	+++	–	SCL	OL	SCL	OL	CL	+	+/+++	OL	–	OL	SCL	CL

## Methods

### Isolates selection and genomic DNA preparation

Four representative isolates of *S.* Typhimurium DT8 and DT30 being used as control positive in Anderson typing scheme of *S.* Typhimurium were selected. Isolates included one DT30 (MS57) and three DT8 (PB225, PB469 and PB880) strains. Bacterial isolates were cultured on nutrient agar media and incubated overnight at 37 °C. Bacterial colonies were removed from the culture plate with an inoculation loop and genomic DNA was extracted using QIAamp^**®**^ DNA Mini kit (Qiagen) according to manufacturer’s instructions. DNA quality and quantity were checked by gel electrophoresis and Qubit^**®**^ quantification platform (Invitrogen) respectively. 20 µl of DNA (20–50 ng/µl) from each isolate was submitted for Illumina sequencing.

### Genomic library preparation and sequencing

Whole genome sequencing (WGS) was performed using an Illumina MiSeq on 250 bp paired-end (PE) libraries. The raw paired fastq sequence data were submitted to European Nucleotide Archive (ENA) http://www.ebi.ac.uk/ena/data/view/PRJEB8262.

Accession numbers are available in Table 2. We also included the whole genome of a clinical isolate of *S.* Typhimurium DT8 (ERS007592) that was isolated from human stool in 2009, UK [37] and the raw paired fastq sequence files for this isolate were downloaded from ENA http://www.ebi.ac.uk/ena/data/view/ERR024405&display=html.

**Table 2:** List of the *S.* Typhimurium isolates used in this study

Isolate	Phage type (DT)	Accession number	References
*S.* Typhimurium MS57	DT30	ERS640854	This study
*S.* Typhimurium PB225	DT8	ERS640855	This study
*S.* Typhimurium PB469	DT8	ERS640856	This study
*S.* Typhimurium PB880	DT8	ERS640857	This study
*S.* Typhimurium DT8	DT8	ERS007592	[38]
*S.* Typhimurium LT2	DT4	AE006468	[18]
*S.* Typhimurium UK-1	DT1	CP002614	[19]
*S.* Typhimurium SL1344	DT44	FQ312003	[20]

### Sequence data quality control

The quality of PE Illumina sequence data for each isolate was evaluated using the FastQC toolkit (http://www.bioinformatics.babraham.ac.uk/projects/fastqc/). Adapter sequences were trimmed and low quality reads were removed using ea-utils package (https://code.google.com/p/ea-utils/).

### Read mapping and de novo sequence assembly

Sequence reads from each isolate were mapped against the reference genome of *S.* Typhimurium strain LT2 (that belongs to phage type 4; DT4) along with its associated plasmid (pSLT) using Burrows Wheeler Aligner (BWA) [38]. Genomic variants including single nucleotide polymorphisms (SNPs) and insertions and deletions (indels) were identified using samtools mpileup [39] and filtered with a minimum mapping quality of 60 (i.e. 1 in 1,000,000 chance of a miss-called variant) are only accepted. Genuine SNPs were present in both forward and reverse direction and supported by ≥70 % of the reads.

The impact of variants were evaluated using SnpEff program (http://snpeff.sourceforge.net/index.html). SNPs were compared to the reference genome of *S.* Typhimurium strain LT2 and a maximum likelihood (ML) phylogeny of the isolates was constructed using MEGA6 software [40]. Selection of the best-fit model for nucleotide substitution was carried out by jModelTest [41].

Reads were de novo assembled using Velvet [42]. The parameters ‘k-mer length, expected coverage, coverage cut-off and insert length’ were optimized to obtain the highest N50 value and the best possible assembly. Generated multi-contig draft genomes for each isolate were analysed and screened for genomic regions and structures that might be associated with phage susceptibility. Genome annotation was performed with the help of Rapid Annotation using Subsystem Technology (RAST) system [43].

## Results

### Identification of SNPs and Indels

Mapping short Illumina PE reads of the DT30 (MS57) isolate to the reference *S.* Typhimurium strain LT2 (phage typed as DT4) revealed 2538 SNPs and 96 indels within the bacterial chromosome. While the four DT8 isolates (PB225, PB469, PB880 and ERS007592) showed 2407, 2431, 2420 and 2096 SNPs respectively and 89, 97, 93 and 101 indels respectively. SNPs were randomly distributed around the bacterial chromosome. Variant call files (VCF) including SNPs and their effect for DT8 and DT30 chromosomes are provided in Additional file 1: Table S1. The majority of the SNPs were silent ranging from 63.2 % in DT8 to 64.5 % in DT30. Nonsense mutations varied from 0.5 % in DT30 to 0.7 % in DT8. The missense mutations ranged from 35 % in DT30 to 36.04 % in DT8. Non-synonymous mutations are involved within the genes of the integrated prophages of the reference *S.* Typhimurium strain LT2 (DT4). Detailed gene-by-gene information is provided in Additional file 2: Table S2.

**Fig. 1:**
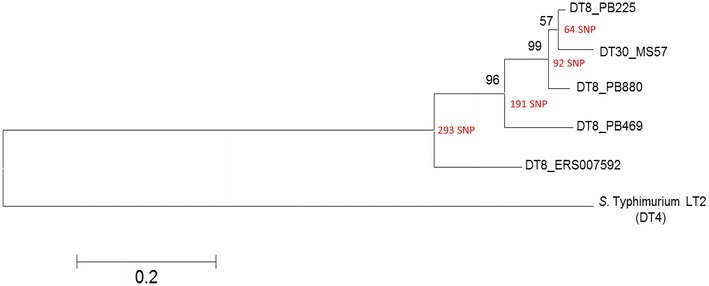
Maximum-likelihood phylogenetic tree of *S.* Typhimurium strains. The tree is based on SNPs determined from the whole genome sequence (excluding prophage regions and phage remnant). Tree was inferred by using a general time-reversible (GTR) model with a gamma distribution. Bootstrap support values, given as a percentage of 1000 replicates, are shown on the branches. SNPs supporting each branch are displayed in *red*

Visualization of the mapped reads and variants for each isolate using Integrative Genomics Viewer (IGV) [8] confirmed that the majority of the SNPs are located within the integrated prophages of the reference *S.* Typhimurium strain LT2. All variants within the prophages were therefore excluded to obtain 885 SNPs within DT30 (MS57) and 953, 939, 847 and 877 SNPs within DT8 isolates PB225, PB469, PB880 and ERS007592 respectively. SNPs within the chromosome (excluding prophages) of DT8 and DT30 isolates are provided in Additional file 3: Table S3. Although there are 716 SNPs common among both DT8 and DT30 isolates DT30 has 33 unique SNPs that do not occur in any of the DT8 strains. ML tree showed close relation among DT30 and DT8 strains with no significant divergence among them as illustrated in Fig. 1.

### De novo assembly

De novo assembly of the 250 bp PE Illumina reads of the *S.* Typhimurium DT8 (PB225) isolate yielded an N50 scaffold size of 275,446 bp; largest scaffold = 660,336 bp with median coverage depth 34×, N50 of 83,098; largest scaffold = 263,713 bp for DT8 (PB469) strain with median coverage depth 15.9×, N50 of 184,411 bp; largest scaffold = 286,128 bp for DT8 (PB880) strain with median coverage depth 34×, N50 of 377,002 bp; largest scaffold = 600,548 bp for the DT8 (ERS007592) strain with median coverage depth 27.5× and N50 of 132,774 bp; largest scaffold = 568,574 bp for the DT30 (MS57) variant with median coverage depth 29×.

### Prophages in *S.* Typhimurium DT8 and DT30

**Fig. 2:**
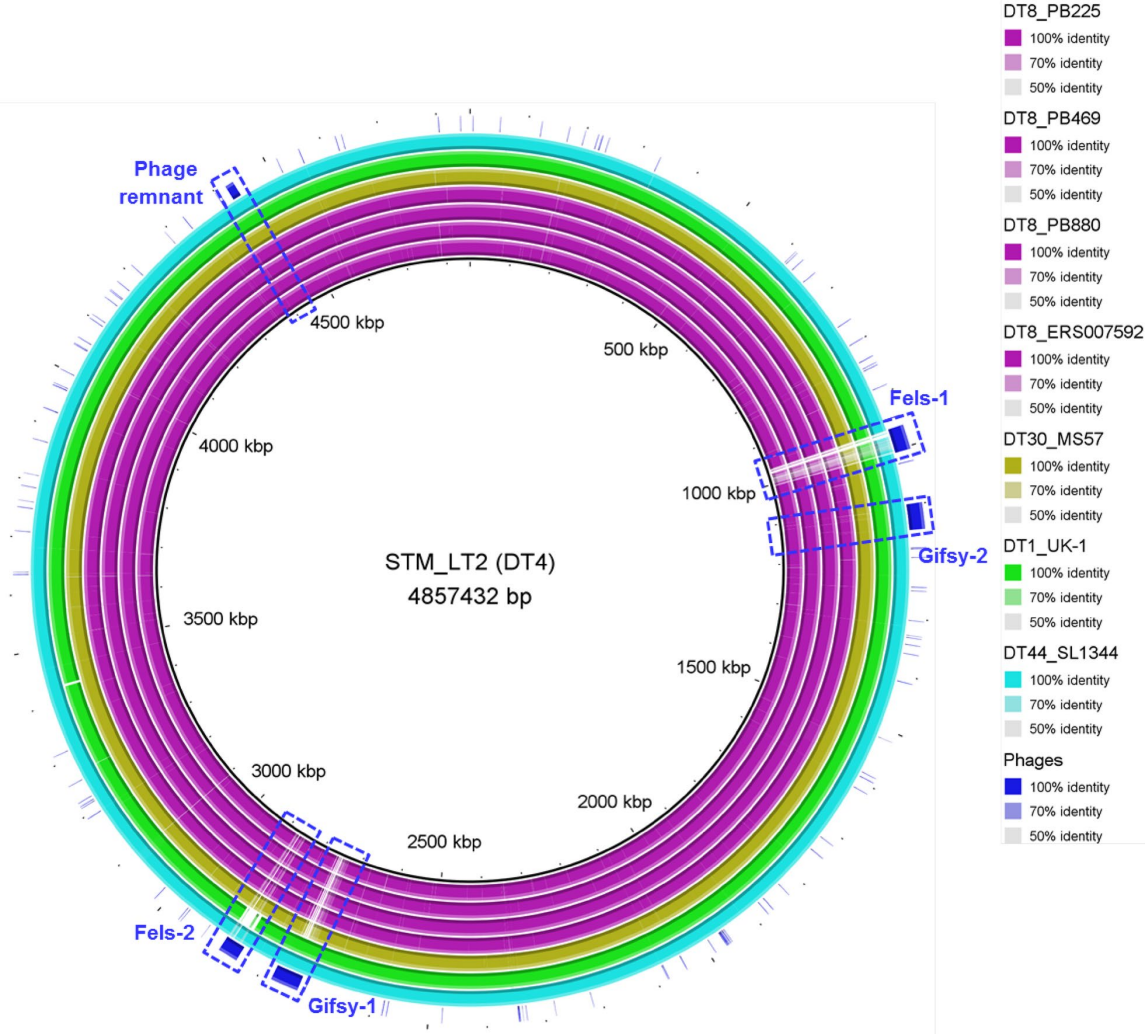
Complete genome alignment of different *S.* Typhimurium phage types generated using BRIG [44]. *S.* Typhimurium strain LT2 (DT4) genome is used as a reference and its four associated prophages (Fels-1, Fels-2, Gifsy-1 and Gifsy-2) and the phage remnant are also included in the alignment. The draft genome of both DT8 and DT30 variants harbours Gifsy-2 and the phage remnant but lacks Gifsy-1, Fels-1 and Fels-2

**Fig. 3:**
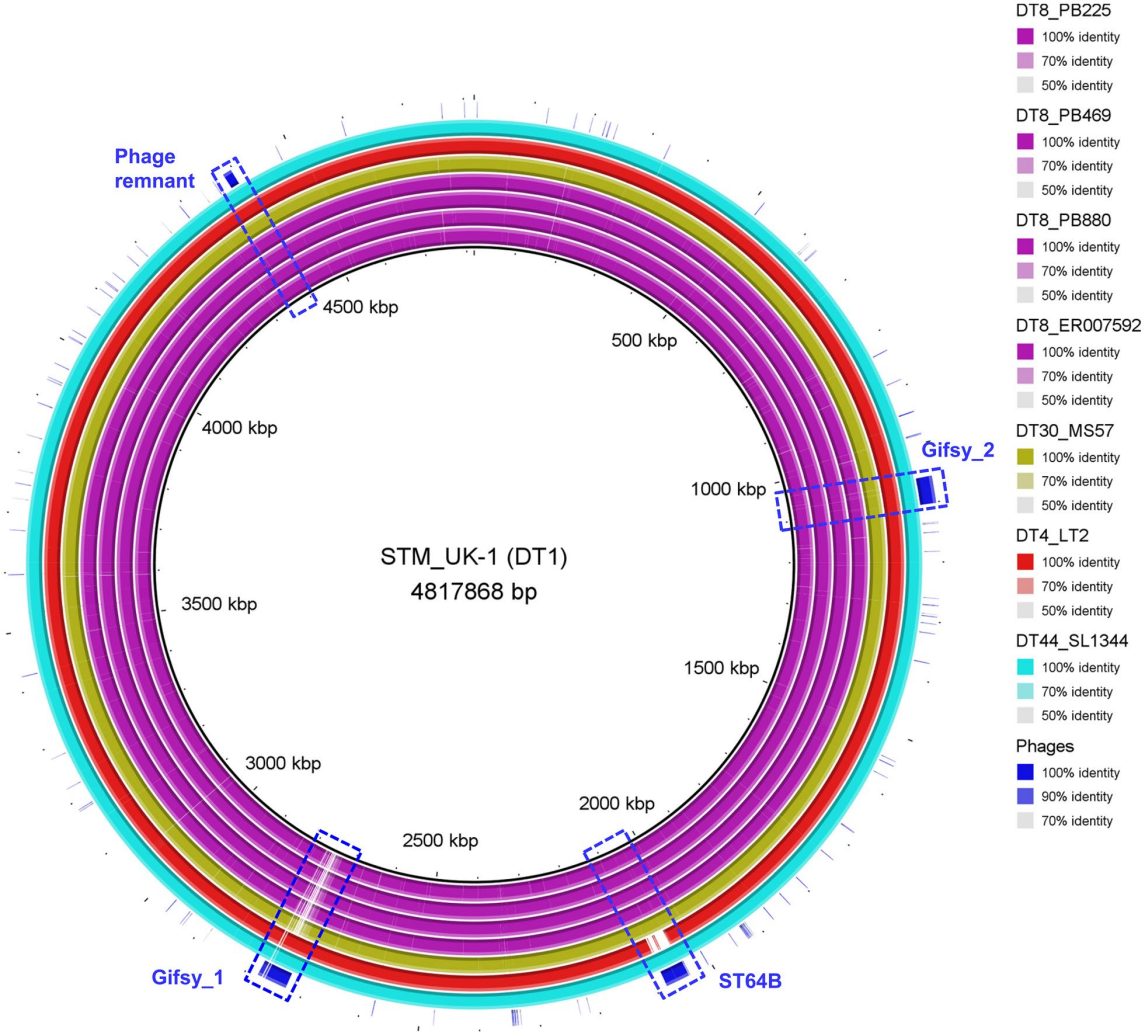
Complete genome alignment of different *S.* Typhimurium phage types generated using BRIG [44]. *S.* Typhimurium strain UK-1 (DT1) genome is used as a reference and its three associated prophages (Gifsy-1, Gifsy-2 and ST64B) and the phage remnant are also included in the alignment. The draft genome of both DT8 and DT30 variants harbours Gifsy-2, ST64B and phage remnant but lacks Gifsy-1 phage

**Fig. 4:**
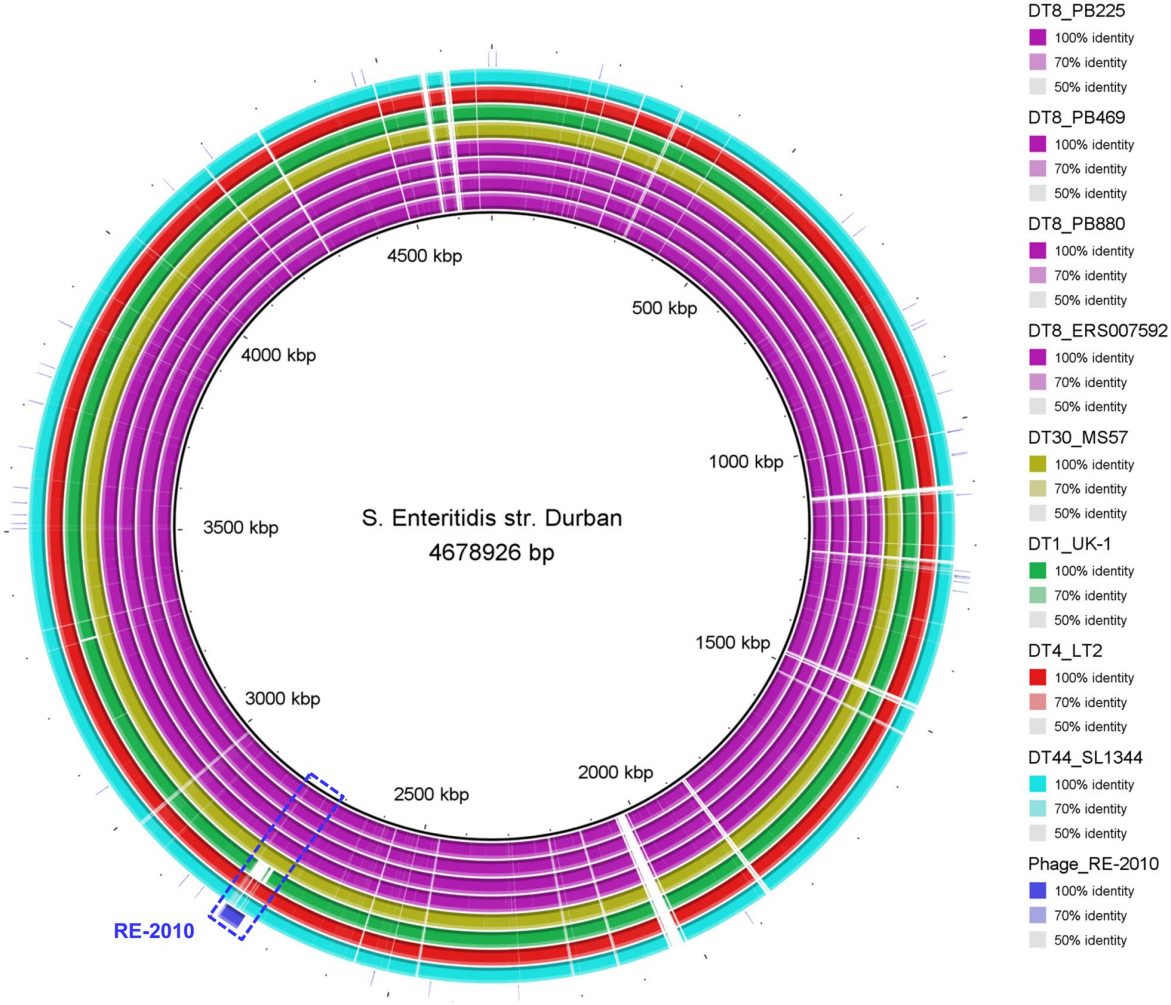
Complete genome alignment of different *S.* Typhimurium phage types generated using BRIG [44]. *S.* Enteritidis strain Durban genome is used as a reference and its associated prophage RE-2010 is also included in the alignment. The draft genome of both DT8 and DT30 variants harbours RE-2010 prophage which is absent from other *S.* Typhimurium strains including *S.* Typhimurium strains UK-1 (DT1). A closely related phage to RE-2010, Fels-2, is present in *S.* Typhimurium LT2 (DT4) and SL1344 (DT44)

The draft genome of both *S.* Typhimurium DT8 and DT30 harbours three prophages as confirmed by PHAST [9] including phage Gifsy-2 (Figs. 2, 3), phage ST64B (Fig. 3) and phage RE-2010 (Fig. 4) as well as a phage remnant (Figs. 2, 3). RE-2010 is a *S.* Enteritidis associated prophage belongs to Enteritidis lysogenic phage S (ELPhiS) which has not been detected previously in any of the *S.* Typhimurium strains.

Mapping short Illumina PE reads of DT8 and DT30 to the reference genomes of phages Gifsy-2 (GenBank accession NC_010393), ST64B (GenBank accession AY055382) and RE-2010 (GenBank accession HM770079) revealed that the sequence of the prophage RE-2010 in both DT8 and DT30 is conserved. The prophage ST64B of DT8 has one coding SNP (at position 18582) located in *sb25* gene coding probable tail fibre protein that does not occur in the ST64B prophage of DT30 however, this SNP was synonymous and did not change the protein sequence. Prophage Gifsy-2 of DT8 has one synonymous SNP (at position 11178) coding bacteriophage damage-inducible protein DinI. Interestingly, prophage antitermination protein (locus_tag: STM1022) of phage Gifsy-2 has two SNPs including one synonymous SNP in DT8 in addition to one non-synonymous SNP in DT30 that changed the amino acid tryptophan (Trp, W) in DT8 to cysteine (Cys, C) in DT30.

### RMS in *S.* Typhimurium DT8 and DT30

*Salmonella* Typhimurium DT8 and DT30 draft genomes contain the four types of restriction-modification systems (RMS); I, II, III and IV as illustrated in Table 3. However, DT8 carries an extra methyltransfearse (MTase) belonging to type II RMS that is closely related (84.25 % identity) to M.EcoGIX MTase. Analysis of the RMS within other strains of *S.* Typhimurium belonging to different phage types including DT1 (strain UK-1), DT4 (strain LT2) and DT44 (strain SL1344) revealed that the closely related M.EcoGIX MTase is carried on the plasmids of DT1, DT4 and DT44, M.SenTFII MTase (type I RMS) is unique to DT4 while absent from other phage types and M.Sen158III MTase (type II RMS) is unique to DT8 and DT30 while absent from DT1, DT4 and DT44.

**Table 3:** List of the four types of RMS present within different *S.* Typhimurium phage types as confirmed by REBASE [44]

RMS genes	Function	Recognition sequence	DT1	DT4	DT44	DT8	DT30
Restriction modification system							
Type I RMS							
*EcoKI*	Restriction enzyme	AACNNNNNNGTGC	+	+	+	+	+
*M.Sen1736III*	Methyltransferase	GAGNNNNNNRTAYG	+	−	+	+	+
*S.Sen318I*	Specificity subunit		+	+	+	+	+
*M.SenTFII*	Methyltransferase	GAGNNNNNNRTAYG	−	+	−	−	−
Type II RMS							
*M.Sen1736V*	Methyltransferase	GATC	+	−	+	+	+
*M.Sen158IV*	Methyltransferase	BATGCATV	+	+	+	+	+
*M.Sen158III*	Methyltransferase	GATC	−	−	−	+	+
*M.SenAboDcm*	Methyltransferase	CCWGG	+	+	+	+	+
*Sen1736II*	Restriction enzyme/methyltransferase	GATCAG	+	+	+	+	+
*M.EcoGIX*	Methyltransferase	SAY	+	+	+	+	−
Type III RMS							
*SenAZII*	Restriction enzyme		+	+	+	+	+
*M.Sen1736I*	Methyltransferase	CAGAG	+	+	+	+	+
Type IV RMS							
*StyLT2Mrr*	Methyl-directed restriction enzyme		+	+	+	+	+

### CRISPRs in *S.* Typhimurium DT8 and DT30

**Fig. 5:**
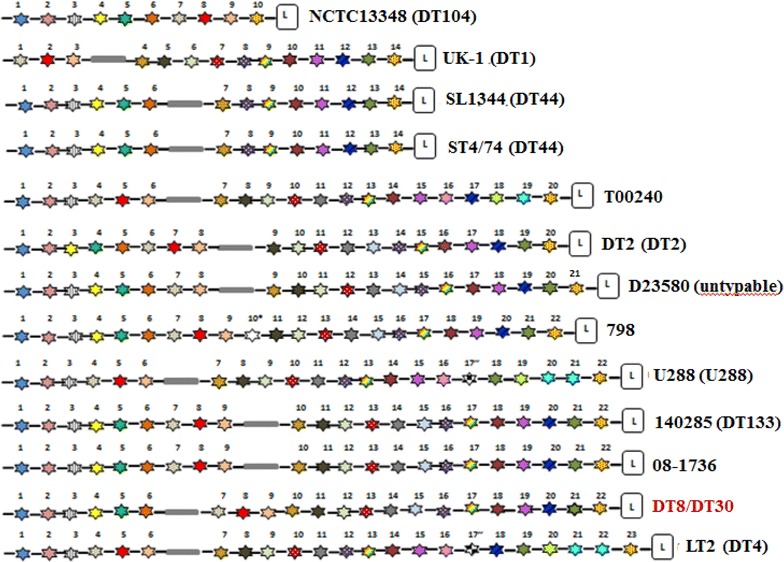
Overview of CRISPR-1 locus in different strains of *S.* Typhimurium. Leader is shown as (L). Repeats are similar and shown as *black lines*. Spacers are shown as *coloured stars*. Spacers with identical sequence are shown in the *same colour*. *White stars* represent strain specific spacers. DT8 and DT30 have identical spacers. Strain 798 has a unique spacer (10*). Strains LT2 (DT4) and U288 (U288) share a unique spacer (17″)

Two CRISPR loci, CRISPR-1 and CRISPR-2, were detected within both *S.* Typhimurium DT8 and DT30 using CRISPRFinder [10]. The two variants, DT8 and DT30, contain highly similar palindromic repeats to other *S.* Typhimurium strains however their spacers are identical. DT8 and DT30 have the smallest number of spacers compared to the other *S.* Typhimurium strains as showed in Table 4 suggesting exposure to fewer phages. Analysis of CRISPR-1 locus among different *S.* Typhimurium phage types showed the variation in the number and pattern of spacers as illustrated in Fig. 5. However, some spacers are located at the same position within *S.* Typhimurium strains which is consistent with the hypothesis of common ancestor.

**Table 4:** Distribution of the CRISPR loci detected in 13 different strains of *S.* Typhimurium

Strain	Phage type	Accession number	Total no. of spacers in CRISPR loci	Spacers no. In CRISPR-1 (CRISFK-1 coordinates)	Spacers no. In CRISPR-2 (CRISPR-2 coordinates)
UK-1	DTI	CP002614	37	14 (3045088–3046075)	23 (3062207–3063639)
DT2	DT2	HG326213	46	20 (3035608–3036960)	26 (3053092–3054707)
lt2	DT4	AE006468	55	23 (3076611–3078147)	32 (3094279–3096260)
SL1344	DT44	FQ312003	37	14 (3099172–3100159)	23 (3116291–3117723)
ST4/74	DT44	CP002487	37	14 (3099172–3100159)	23 (3116291–3117723)
NCTC13348	DT104	HF937208	36	10 (3111855–3112493)	26 (3128625–3130239)
14028S	DT133	CP001363	48	22 (3096848–3098323)	26 (3114455–3116070)
08–1736	–	CP006602	48	22 (4332390–4333865)	26 (4349997–4351612)
U288	U288	CP003836	54	22 (3074262–3075737)	32 (3091869–3093850)
798	–	CP003386	44	22 (3097927–3099324)	22 (3115456–3116827)
D23580	Untypable	FN424405	39	21 (3069598–3071012)	IS (3087144–3088271)
T000240	DT12	AP011957	52	20 (3100041–3101393)	32 (3117525–3119506)
PB225	DT8	ERS640E55	31	22	9
MS57	DT30	ERS640854	31	22	9

Each strain (phage type) contains a variable number of spacers and both DT8 and DT30 have the lowest number of spacers

SL1344 and ST4/74 strains contain identical spacers and both belong to the same phage type (DT44)

UK-1 (DT1) has the same number of spacers as SL1344 (DT44) and ST4/74 (DT44), however they belong to a different phage type as DT1 has 6 unique spacers that do not occur in DT44 variants

(–) indicates that phage type is not known

### Novel mobile genetic elements in *S.* Typhimurium DT8 and DT30

**Fig. 6:**
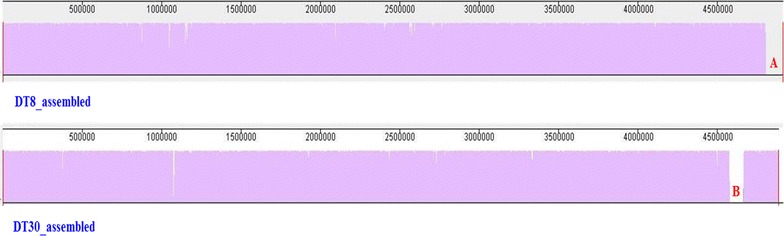
Draft genome alignment of *S.* Typhimurium DT30 (MS57) and a representative DT8 (PB469) generated by progressiveMauve [45]. Conserved regions are coloured in purple. The unaligned sequence element (**a**) represents a pSLT related plasmid (GenBank accession AE006471) and it is unique to DT8 with no detectable homology in DT30. The unaligned sequence element (**b**) represents an integrative and conjugative element and it is unique to DT30 with no detectable homology to DT8

The four representative *S.* Typhimurium DT8 draft genomes harbour a plasmid (~93 kb) that is genetically related to the virulence pSLT plasmid of *S.* Typhimurium strain LT2. Two plasmid replicons were identified in DT8 draft genomes using PlasmidFinder [11]. DT8 specific plasmid differs from the pSLT plasmid in 13–17 SNPs and 1–4 indels (total variants are illustrated in Additional file 4: Table S4). DT8 specific plasmid has four coding nonsynonyms SNPs in locus tags; pSLT014, pSLT057 and pSLT079 coding putative outer membrane protein, putative cytoplasmic protein, conjugal transfer pilus assembly protein (*traE*) respectively. Although DT30 lacks the pSLT related plasmid it carries a distinctive chromosomal mobile element absent from DT8 as illustrated in Fig. 6. This element (~60 kb) identified by BLAST [12] as an integrative and conjugative element (ICE) that is genetically related to the ICE (~75 % identity) of the Enterotoxigenic *Escherichia coli* (ETEC) strain UMNK88 (GenBank accession CP002729) [13]. This novel mobile element encodes a type IV secretion pathway system (T4SS) as confirmed by Pfam [14] and several hypothetical proteins of unknown function (annotation of the ICE is provided in Additional file 5: Table S5).Analysis of the complete genome of other *S.* Typhimurium strains including UK-1 (DT1) and SL1344 (DT44) revealed the absence of ICE but the presence of a plasmid closely related to the pSLT plasmid of *S.* Typhimurium strain LT2 that has been also detected in *S.* Typhimurium DT8 but missing from DT30.

## Discussion

Phage typing has been used for more than four decades as a rapid, low cost approach in epidemiological characterization of *S.* Typhimurium however, the underlying molecular basis of phage typing is not well described. Although phage typing has certain limitations [15] it is still in use and represents a convenient model for studying phage-host interactions. In the *S.* Typhimurium phage typing scheme, bacterial strains are classified into more than 300 DTs based on the pattern of their sensitivity or resistance to a set of the typing phages. Interestingly, DT8 and DT30 have distinct pattern in phage susceptibility (DT30 is resistant to all typing phages except phage 8 whereas DT8 is susceptible to eleven phages) although both phage types were reported to be associated with a single foodborne salmonellosis outbreak in Ireland between August 2009 and February 2011. Here we apply WGS technology to investigate the genomic correlates of the striking difference in phage susceptibility among *S.* Typhimurium phage types, DT8 and DT30.

Bacterial antiphage systems include blockage of phage DNA entry through a phenomenon known as ‘Sie’ where existing prophage within bacterial genome prevents infection by the same or closely related viruses [16]. Integrated prophage can block phage DNA penetration into bacterial cell as it codes blocking proteins that are localized at the membrane/cell wall level of the host cell. A strong relation between the integrated prophages and the phage type was observed [17]. For example, *S.* Typhimurium strain LT2 contains 4 prophages; (Fels-1, Fels-2, Gifsy-1 and Gifsy-2) [18], *S.* Typhimurium strain UK-1 contains 3 prophages, two that are homologous to those in LT2 (Gifsy-1, Gifsy-2) in addition to ST64B prophage [19] and *S.* Typhimurium strain SL1344 contains 4 prophages; 3 homologs of those in LT2 (Gifsy-1, Gifsy-2, Fels-2) and ST64B [20] and each of these strains has a distinct typing pattern as LT2, UK-1 and SL1344 strains belong to DT4, DT1 and DT44 respectively. However, in this study we found that both *S.* Typhimurium DT8 and DT30 contain the same 3 prophages. Two prophages are associated with *S.* Typhimurium including Gifsy-2 (also present in DT4) and ST64B (detected also in DT1). In addition, RE-2010, an *S.* Enteritidis associated prophage, that is closely related to Fels-2 phage [21]. This study is the first to report the presence of ELPhiS within *S.* Typhimurium strains.

Interestingly, prophage Gifsy-2 of DT30 has a unique non-synonymous SNP in the prophage antitermination protein that changed the amino acid tryptophan (Trp, W) in DT8 to cysteine (Cys, C) in DT30. Replacement of tryptophan by cysteine could negatively affect the function of antitermination protein since tryptophan is a unique amino acid in terms of chemistry and size.

CRISPR-Cas system has also been described as one of the prokaryotic antiviral defence systems [22]. It is considered as the adaptive bacterial immune system that provides acquired immunity against foreign DNA through targeting invading DNA in a sequence-specific manner. CRISPRs are acquired from invading phages and/or plasmids and incorporated within bacterial genome. These palindromic repeats are interspaced by spacers (interspaced regions between palindromic repeats) which are useful in providing information on the past exposure of the bacteria to foreign DNA including phages and/or plasmids. The immunization process is based on neutralizing foreign DNA through a mechanism similar to RNA interference (RNAi) [23]. It is considered that a strain with more spacers is consistent with exposure to more phages and DNA invasion along the lineage of that strain. However, a recent study suggested that *Salmonella* CRISPR-Cas systems are not immunogenic anymore [24].

CRISPRs were found to be variable among different *S.*Typhimurium phage types [19] and a strong correlation between CRISPR and phage type was reported and it has been suggested that CRISPR typing might be a powerful laboratory method for surveillance of *Salmonella* infections [25]. However, the presence of identical palindromic repeats interspaced with identical spacers among *S.* Typhimurium DT8 and DT30 analyzed in this study revealing the limitations of CRISPR typing for *Salmonella* surveillance.

It has been reported that genetic diversity among different phage types of *S.* Typhimurium is mainly due to accumulation of SNPs [26] which can result in gene inactivation. Interestingly, SNP typing of *S.* Typhimurium has been recommended to have the potential to replace the phage typing scheme [27]. However, our phylogenetic analysis based on SNPs determined from WGS of DT8 and DT30 did not reveal significant genetic divergence among the two phage types and SNPs were randomly distributed around bacterial chromosome. In fact, the historical control DT8 isolates (PB225, PB469 and PB880) and DT30 isolate (MS57) are more closely related to each other than to the recently isolated clinical DT8 isolate (ERS007592) suggesting that DT30 might have arisen from DT8.

RMS allows bacteria to recognize and destroy viral DNA by restriction endonucleases (REases) that have the ability to cut foreign DNA at certain sequence (restriction sites) while bacterial DNA is protected by the aid of DNA MTase that modifies restriction sites within the bacterial genome [28, 29]. *S.* Typhimurium DT8 and DT30 contain similar RMS however DT8 has an extra MTase that is very similar to M.EcoGI (84.25 % identity). M.EcoGIX MTase belongs to type II RMS and is a very unusual kind of MTase as it methylates only one of the double strands of DNA and it can modify adenines in a wide variety of sequence contexts [30]. Recently, it has been shown that M.EcoGIX MTase plays an important role in protecting the harbouring plasmid from digestion by host-encoded REases and subsequently expanding the plasmid host range [31]. We speculate that M.EcoGIX related MTase may play a similar role in protection of phage DNA thus allowing phage to multiply within bacterial cells. Several *Bacillus subtilis* phages incorporate modified bases into their genome [32, 33] however it is not known if this is true for the *S.* Typhimurium typing phage. It is also possible that M.EcoGIX related MTase may be required for transcription of certain phage genes ensuring phage replication as reported earlier [34]. We note however that the methyl-directed REase (StyLT2Mrr) within the chromosome of both DT8 and DT30 appears to have the ability to cleave modified bases.

M.EcoGIX related MTase is carried on the plasmid of *S.* Typhimurium DT8 strains. The plasmid is genetically related to the pSLT plasmid of *S.* Typhimurium strain LT2 (DT4). Closely related plasmids to pSLT including pSTUK-100 and pSLT-SL1344 were also detected within *S.* Typhimurium strains UK-1 (DT4) and SL1344 (DT44) respectively. Acquisition of these closely related plasmds might be linked with the differences in phage susceptibility since an earlier study showed the change between phage types in *S.* Enteritidis was related to acquisition of a plasmid [35].

*Salmonella* Typhimurium DT30 does not carry any accessory genomes including pSLT related plasmid but it harbours a unique genetic mobile element, ICE, which is absent from the DT8 strains as well as other phage types including DT1, DT4 and D44. Sequence analysis of specific DT30 ICE did not reveal the presence of any REases or MTases that might be associated with bacterial resistance to almost all phages used in the typing scheme but ICE harbours T4SS and several orphan genes coding hypothetical proteins (proteins of unknown functions) that might play a role in bacterial reaction to bacteriophages. Few reports showed that ICE might harbour genes allowing bacteria to grow in the presence of harsh environment containing antibiotics, heavy metals as well as bacteriophages [36, 37]. However additional studies including prediction of the structure and function of ICE hypothetical proteins within DT30 are required in order to evaluate the role of ICE in bacterial resistance to phages.

The high similarity among the genome of DT8 and DT30 at the whole genome level does not explain the distinct differences in phage susceptibility among the two phage types. However, we suggest that the acquisition of novel genetic elements including the pSLT like plasmid in DT8 (absent from DT30) and/or the ICE in DT30 (absent from DT8) are significant in the distinct difference in phage susceptibility among the two phage types. Further experimental work is required to explore the hypothesis generated by this project which may have broader relevance in understanding phage-host interactions.

## Conclusions

*Salmonella* Typhimurium phage typing scheme represents a good model for studying phage-host interactions. Comparative genomic analysis of the two *S.* Typhimurium DT8 and DT30 associated with a single foodborne outbreak represents an initial step toward understanding genetic basis of this *S.* Typhimurium phage-host interaction. The great difference in phage susceptibility between DT8 and DT30 and the identification of discrete points of differences in otherwise very similar strains makes this a useful model for further study of phage-host interaction. Additional studies are required to determine the significance of the differences among DT8 and DT30 in relation to the difference in phage susceptibility. Understanding the molecular basis for phage-host interactions may ultimately lead to engineering of lysogenic bacteriophage with specific spectra that may be used for treatment of resistant bacterial infections or for delivering antimicrobial agents to resistant bacteria.

## Additional files

10.1186/s13104-015-1687-6 VCFs of DT8 and DT30 including SNPs and their effect.
10.1186/s13104-015-1687-6 Gene-by-gene information for DT8 and DT30 variants.
10.1186/s13104-015-1687-6 SNPs within the chromosome (excluding prophages) of DT8 and DT30 isolates.
10.1186/s13104-015-1687-6 Total variants among pSLT plasmid and the DT8 specific plasmid.
10.1186/s13104-015-1687-6 Annotation of the integrative and conjugative element of the DT30 isolate.

## Abbreviations

CRISPR: clustered regularly interspaced short palindromic repeats
DT: definitive phage type
ENA: European nucleotide archive
ETEC: enterotoxigenic *Escherichia coli*
ICE: integrative and conjugative element
IGV: integrative genomics viewer
Indels: insertions and deletions
MTase: methyltransferase
REase: restriction endonuclease
RMS: restriction-modification system
Sie: SUPERINFECTION exclusion
SNP: single nucleotide polymorphism
*S.* Typhimurium: *Salmonella**enterica* subsp. *enterica* serovar Typhimurium
T4SS: Type IV secretion pathway system
VCF: variant call file
WGS: whole genome sequencing
